# Out-of-hospital cardiac arrests in children

**DOI:** 10.4103/0974-2700.66531

**Published:** 2010

**Authors:** Antti Kämäräinen

**Affiliations:** Helsinki Area Helicopter Emergency Medical Service Medi-Heli 01, Vantaa, Finland, Critical Care Medicine Research Group, Tampere University Hospital, Tampere, Finland

**Keywords:** Cardiac arrest, children, CPR, pediatric, prehospital, resuscitation, out-of-hospital

## Abstract

Prehospital pediatric cardiac arrest is a rare event compared with adult cardiac arrest. Despite the recent advancements in postresuscitation care improving the outcome of adult patients, similar evidence is lacking in pediatric victims of cardiac arrest. In this brief article, the current data on pediatric cardiac arrest occurring in the prehospital setting are reviewed. The annual incidence of pediatric out-of-hospital cardiac arrest is approximately 8–10 cases per 100,000 persons. The outcome is generally poor, as only 2–9.6% of patients survive to hospital discharge. The neurologic outcome of survivors is good in 24–31% of patients. Current evidence is insufficient to strongly support or refute the use of mild therapeutic hypothermia during the postresuscitation phase in pediatric patients. The application of a goal-directed treatment protocol for pediatric cardiac arrest and postresuscitation syndrome needs to be evaluated.

## INTRODUCTION

There are few other emergencies that cause as much discomfort to the prehospital emergency medical service (EMS) personnel than pediatric cardiac arrest and resuscitation. Indeed, not only are these events rare and resuscitation of the pediatric patient regarded as stressful, but also the outcome of this condition is often dismal.[1–3] Recent advances in resuscitation science, such as the application of mild therapeutic hypothermia in the postresuscitation phase has improved the outcome of adult cardiac arrest victims.[4,5] Similar evidence, however, is lacking in the pediatric population.

The purpose of this review was to briefly summarize the contemporary aspects of prehospital pediatric cardiac arrest and resuscitation. Epidemiologic aspects, such as the incidence, etiology, and outcome are evaluated, as well as the unique features of pediatric cardiac arrest and resuscitation. Articles were searched using the PubMed and MEDLINE databases with the following search terms: “Heart arrest,” “Cardiac arrest,” “Resuscitation,” or “CPR,” and “Pediatric,” “Paediatric,” or “Children,” and “Out-of-hospital,” “Out of hospital,” or “Prehospital.” Articles were considered relevant when the incidence and outcome rates were clearly reported and etiologies and neurologic outcome at hospital discharge assessed.

### Epidemiological aspects

During the past 20 years, the annual incidence of pediatric prehospital cardiac arrest has constantly remained at 8.0–9.8 cases per 100,000 persons.[3,6,7] The arrest occurs most commonly (88%) in a nonpublic location, such as the residence.[3] The mean age of the patients ranges between 2.9 and 6.2, and male patients represent a slight majority (53–62%).[3,6,7] The most common etiologies of arrest have been reported to be sudden infant death syndrome, trauma, and respiratory.[2,6] Still, it is not uncommon that there is no obvious cause for the arrest identifiable in the initial resuscitation phase.[2] Thus it may well be impossible to determine the cause of arrest in at least 14.5% of the cases[8] until autopsy is performed.[7] Also, there is evidence indicating that prehospital clinical diagnosis on the cause of pediatric cardiac arrest has low agreement when compared with coroner’s postmortem diagnosis.[8] The most frequently unanticipated diagnoses have been reported to be pneumonia and seizure-related cardiac arrest.[8]

The majority (66–77%) of arrests are not witnessed[2,3,6,7,9] and the bystanders initiate cardiopulmonary resuscitation (CPR) in only 23–35% of cases.[2,3,6,7] Bystander use of an automated external defibrillator is practically nonexistent.[3] The most common initial cardiac rhythm is nonshockable (82%),[3] more specifically asystole in 77.2–79% and pulseless electrical activity in 13.5–16.4% of arrests.[6,7] As in the adult population, asystole as the initial cardiac rhythm predicts worse survival than other initial rhythms, especially those of shockable nature.[2]

The overall survival to hospital discharge after pediatric prehospital cardiac arrest is low with the reported percentages of 2.0–9.6%.[2,3,6,7,10] Survival to hospital discharge is more common among children and adolescents than in infants.[3] One study has calculated the number needed to treat (NNT) in different age groups. In that study, the NNT for survival to hospital discharge was 13 for both pediatric and adult victims of cardiac arrest, whereas more specifically NNT was 29 for infants (<1 year), 10 for children (age, 1–11 years), and 8 for adolescents (age, 12–19 years).[3] The neurologic outcome at hospital discharge according to the pediatric cerebral performance category (PCPC)[11] is good (PCPC, 1–2) in 24–31% of the cases.[2,12]

### Pediatric resuscitation

**Figure 1:**
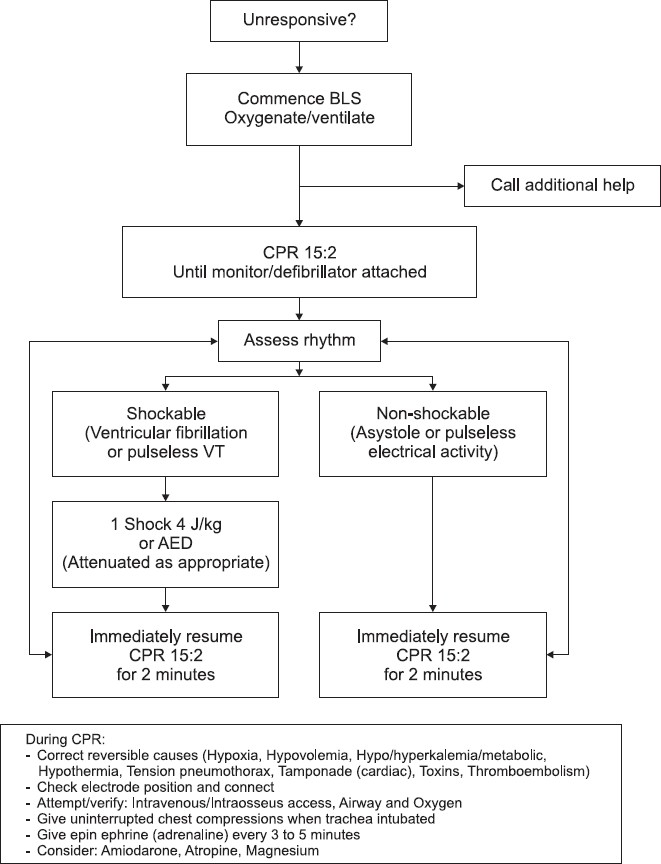
Figure 1: Pediatric advanced life support algorithm, modified from the 2005 resuscitation guidelines.[14] BLS, basic life support; CPR, cardiopulmonary resuscitation; VT, ventricular tachycardia; AED, automated external defibrillator

The current pediatric basic life support (BLS) and advanced life support (ALS) [Figure 1] guidelines were published in 2005 and the forthcoming guidelines will be published on October 18, 2010.[13] The unique features of pediatric BLS are the application of 5 initial rescue breaths prior to chest compressions reflecting a response toward a probable respiratory etiology of arrest. Also, in comparison to the adult BLS sequence, a lone rescuer should perform CPR for 1 min prior to seeking additional help.[13] Although the American Heart Association recommends compressions-only CPR in adult patients, the pediatric CPR algorithm still includes rescue breaths to be performed.[14] In children, the energy used for defibrillation is weight adjusted rather than fixed, using an energy of 4 J/kg with both monophasic and biphasic defibrillators.[13] Vascular access may be difficult to gain, especially in infants. Thus after 3 failed attempts at intravenous access, an intraosseal needle should be inserted.[13] However, one study has indicated that ALS does not increase survival in comparison to BLS, and also decreased survival was observed in association with increased use of intravenous epinephrine.[10]

After successful resuscitation and restoration of spontaneous circulation, treatment is directed toward stabilization of the postarrest myocardial dysfunction and temperature control.[13] The evidence regarding the optimal treatment of the postresuscitation syndrome and temperature control in pediatric patients is scarce. A recent report could not clearly support nor refute the use of mild therapeutic hypothermia treatment in pediatric patients,[15] and implementation of the treatment has remained low.[16] The current evidence on therapeutic hypothermia in pediatric patients is reviewed elsewhere in this symposium of the journal.

## DISCUSSION

It is imperative to recognize the recently published aspect that pediatric patients suffering a cardiac arrest in the prehospital setting differ greatly from those patients requiring resuscitation in the hospital.[12] For instance, asystole as the initial cardiac rhythm is more common in prehospital cardiac arrest. Mortality is higher in prehospital cardiac arrest, with 70% of this attributed to neurologic causes in contrast to only 20% in-hospital.[12] These differences have to be taken into account when assessing pediatric cardiac arrest studies and planning future clinical trials.

As the outcome from pediatric cardiac arrest is poor, it would be preferable to recognize and prevent conditions, such as asphyxiation, that lead to cardiac arrest.[13] However, in the prehospital setting, the onset of arrest is often unwitnessed,[2,7,9] thus making prevention unlikely. As a secondary measure, the rate of bystander CPR needs to be increased. The simplification of the pediatric BLS algorithm has strived to lower the threshold to initiate CPR.[13] However, bystander CPR is provided at best in only one third of the cases. The current guidelines encourage that the adult BLS sequence also may be used in pediatric cardiac arrest victims rather than do nothing due to the unfounded fear of doing harm.[13]

Based on the studies on adult patients, high-quality continuous CPR needs to be provided by EMS personnel to improve the outcome.[17–19] After successful resuscitation, care must be taken to avoid hyperthermia, as this is detrimental in the postresuscitation phase.[13] Whether the beneficial effects of mild hypothermia observed in adult cardiac arrest victims[4,5] and also after neonatal asphyxia[20] apply to pediatric cardiac arrest victims is yet to be shown. Additionally, a standardized treatment protocol including interventions, such as early percutaneous coronary intervention, therapeutic hypothermia, and control of hemodynamic and metabolic factors has been shown to improve the outcome of adult victims of prehospital cardiac arrest.[21] It is tempting to assume that a similar goal-directed treatment protocol adjusted to pediatric cardiac arrest and postresuscitation syndrome would be beneficial and therefore another likely focus of future research.

Finally, although improved survival might be achieved with the above-discussed interventions, it may be necessary to reconsider attempting resuscitation in certain cases of pediatric cardiac arrest. Although it is considered stressful to withhold or cease resuscitation attempts in children,[1] such an approach might be necessary, especially in cases of unwitnessed arrest with asystole as the initial rhythm as futility has been observed in adult victims in similar conditions[22] and children seem to have no better tendency to recover.[13]

## CONCLUSION

Prehospital pediatric cardiac arrest exerts dismal outcome with regard to the proportion of survivors per EMS-attended resuscitation attempts. This may well reflect the negative initial setting in majority of the cases, that is, unwitnessed arrest and a nonshockable initial rhythm. While the management of pediatric cardiac arrest needs to be further developed and studied regarding, for instance, the application of therapeutic hypothermia, it might also be necessary to discuss the futility and justification of resuscitation in some cases. However, it is important to recognize the differences between prehospital and in-hospital pediatric patients, as well as the distinct characteristics of resuscitating a child compared with an adult victim of cardiac arrest.
